# The multiple mechanisms and modes of cell death after acetaminophen overdose

**DOI:** 10.37349/edd.2025.100569

**Published:** 2025-04-07

**Authors:** Hartmut Jaeschke, Anup Ramachandran

**Affiliations:** Department of Pharmacology, Toxicology & Therapeutics, University of Kansas Medical Center, Kansas City, KS 66160, USA

**Keywords:** Drug hepatotoxicity, acetaminophen, acute liver failure, apoptosis, ferroptosis, lipid peroxidation, inflammation

## Abstract

Acetaminophen (APAP)-induced liver injury and acute liver failure is a significant clinical problem worldwide; in addition, APAP overdoses in animals or in cell culture are used as popular models to study drug-induced liver injury mechanisms and test therapeutic interventions. Early assumptions that APAP toxicity is caused by a single mechanism resulting in a defined mode of cell death in hepatocytes had to be questioned when over the years many different mechanisms and modes of cell death were reported. Although many of the contradictory results and conclusions reported over the years can be attributed to lack of understanding of established mechanisms, methodological problems, and misinterpretation of data, it is increasingly recognized that some of the reported differences in signaling mechanisms and even a switch in the mode of cell death can be caused by variations in the experimental conditions. In this review, examples will be discussed how experimental conditions (dose, solvent, etc.), the experimental system (species, strain, and substrain in vivo, cell type, and in vitro conditions), and also adaptive responses and off-target effects of genetic manipulations and chemical interventions, can impact the mechanisms of cell death. Given that the conditions will determine the results, it is therefore of critical importance to keep in mind the translational aspect of the experiments, i.e., the conditions relevant to the human pathophysiology. Only the full appreciation of these issues will lead to reproducible and clinically relevant results that advance our understanding of all facets of the human pathophysiology and identify clinically relevant therapeutic targets.

## Introduction

Acetaminophen (APAP) is one of the most used analgesic and antipyretic drugs worldwide. In the United States, it is estimated that 45–60 million patients take APAP on a weekly basis [1, 2]. Although considered safe at therapeutic doses, overdoses can cause liver injury and acute liver failure [1, 3]. Because of the presence of APAP in numerous prescription and over-the-counter medications, intentional and unintentional overdosing occurs regularly. In fact, 60,000–80,000 emergency department visits, 30,000–40,000 hospitalizations with > 5,000 patients developing severe liver injury, and 300–500 patients die annually in the United States due to APAP overdose [2, 4, 5]. APAP overdose is the most frequent etiology of acute liver failure in the United States, the United Kingdom, and many other mainly western countries [6]. The clinical relevance of APAP toxicity makes understanding mechanisms of cell death induced by this drug critical for the identification of therapeutic targets and discovery of antidotes. The classical example of this was the recognition that an APAP overdose in the mouse causes the formation of a reactive metabolite, which first depletes glutathione (GSH) and then binds to sulfhydryl groups of proteins triggering cell death [7–9]. This mechanistic insight led to the discovery of the GSH precursor *N*-acetylcysteine (NAC) as an effective antidote against APAP toxicity in mice and in patients [10, 11]. NAC was subsequently approved as a clinical antidote in the 1980s and has since then saved numerous lives of overdose patients [10]. A caveat is that NAC has a narrow therapeutic window and limited efficacy after very high overdoses [12, 13]. Thus, the quest continues to better understand the mechanisms of APAP-induced cell death, acute liver failure, and regeneration. While mechanistic understanding has advanced, countless studies over the last few decades also brought up many controversies including the identification of specific targets for protein binding and its relevance to toxicity, the role and sources of oxidant stress and lipid peroxidation (LPO), and the importance of inflammation as a mechanism of injury and recovery. In addition, with the recognition of various modes of cell death, the original necrosis or oncotic necrosis [7–9] was challenged to be apoptosis [14, 15], necroptosis [16, 17], and lately ferroptosis [18, 19] and even pyroptosis [20, 21]. These contradictory data and conclusions are confusing and difficult to reconcile. Although some of these controversies are clearly based on methodological problems and plain misinterpretation of the data, more recent studies demonstrate that multiple signaling mechanisms dependent on the experimental conditions in animals or specific circumstances in patients, can lead to different modes of APAP-induced cell death, which is the topic of this critical review, which is based on our extensive experience with this topic and the pertinent literature.

## Protein binding and oxidant stress

After the first clinical report of APAP toxicity [22], investigators at the US National Institute of Health developed a mouse model of APAP-induced hepatotoxicity, which mimicked the serious liver injury observed in humans [7–9]. The key mechanistic concept derived from these mouse studies was that the cellular toxicity was caused by a cytochrome P450-derived reactive metabolite, which was typically detoxified by GSH, but after the depletion of cellular GSH levels during an overdose, the reactive metabolite binds to cellular proteins thereby causing cell death [7–9]. Though subsequent events after protein binding were cause for controversy initially (detailed in the section on ferroptosis), progress in resolving this issue was made when it was shown that an oxidant stress was not observed during metabolism of APAP [23, 24] but only after the drug metabolism phase was completed, and that the oxidant stress was located within mitochondria [25, 26]. Moreover, the cell death is critically dependent specifically on mitochondrial protein adduct formation [27, 28] and scavenging of mitochondrial reactive oxygen species (ROS) and peroxynitrite after the protein binding occurs effectively protects against cell death [29–32]. These observations strongly support the current hypothesis that reactive metabolite formation and protein binding especially in mitochondria are the key initiating events in the toxicity, which is then the trigger for a moderate mitochondrial oxidant stress with release of superoxide by complex III towards the cytosol without compromise of mitochondrial respiration [33]. This oxidant stress causes activation of a MAP kinase cascade in the cytosol ultimately leading to phosphorylation of c-jun N-terminal kinase (P-JNK), translocation of P-JNK to the mitochondria [34], and binding to the anchor protein Sab [35] for amplification of the mitochondrial oxidant stress and peroxynitrite formation in the mitochondrial matrix [33, 36]. Finally, the oxidative stress and protein nitration causes the opening of the mitochondrial membrane transition pore (MPTP) leading to the collapse of the membrane potential and cessation of ATP synthesis, which is the cause of cell death [37, 38]. Thus, both processes, i.e., protein adducts formation and the oxidant stress, are essential features of APAP-induced cell death and depend on each other. As was pointed out, there is no toxicity without protein binding but there can be protein binding without toxicity [39]. The latter scenario points to the essential role of oxidant stress as an amplifying process of cell death signaling. Importantly, studying these sequential events is not possible when mechanistic parameters are only measured at a single time point after APAP overdose. The assessment of these parameters at various time points after APAP is critical. Furthermore, when genes or pathways are being modulated to study mechanisms, attention needs to be paid to the possibility that this may cause a shift in the mode of cell death.

## Apoptosis versus necrosis in APAP-induced cell death

APAP-induced cell death is characterized by cell swelling, karyolysis, and release of cell contents. It was generally termed oncotic necrosis. However, with the recognition of apoptosis and its detailed signaling mechanisms, reports were published suggesting that APAP also triggers apoptotic cell death [40, 41]. However, a more detailed assessment of the morphology of the cell death revealed no evidence for the characteristics of apoptosis such as cell shrinkage, chromatin condensation, and the biochemical hallmark of caspase activation [42]. In addition, pancaspase inhibitors, which are highly effective against TNF- or Fas receptor-induced apoptosis [43, 44], did not protect [42]. Thus, under normal circumstances, there is no relevant apoptosis after an APAP overdose in mice [42] or human livers [45, 46]. However, there are some confusing observations. Parameters that are considered part of the apoptosis signaling pathway, are detected in APAP-induced cell death. For example, the TUNEL assay, which detects DNA strand breaks [47], stains necrotic cells during APAP hepatotoxicity [42, 48]. In addition, Bid cleavage and the translocation of both Bax and cleaved Bid to mitochondria are involved in the early outer mitochondrial membrane permeabilization after an APAP overdose [49–52]. Intermembrane proteins released from mitochondria through Bax/Bid pores, e.g., apoptosis inducing factor (AIF) and endonuclease G, translocate to the nucleus and cause DNA fragmentation [53]. Other intermembrane proteins released into the cytosol such as cytochrome c and Smac/DIABLO, do not promote apoptosis because APAP-induced mitochondrial dysfunction and oxidant stress prevents any apoptotic signaling in hepatocytes [54, 55]. Nevertheless, these parameters, which are obviously not specific for apoptosis [15], have been frequently used as evidence for apoptosis in APAP pathophysiology during the last 15 years. However, since no efforts are made to explain where the alleged apoptotic cells are located and the TUNEL assay always shows TUNEL-positive cells in the area of necrosis [56], it appears that these parameters are mainly being used to increase the amount of data in the manuscript rather than as a convincing mechanistic argument for apoptotic cell death [57]. One hypothesis that is sometimes brought up is that APAP-induced cell death might be caused by apoptosis, which rapidly deteriorates into secondary necrosis [11]. However, during a situation where massive apoptosis cannot be completed and converts into secondary necrosis with cell swelling and cell contents release, the extensive caspase activation of the primary apoptosis is still measurable [44]. Thus, there is also no evidence for apoptosis switching to secondary necrosis during APAP hepatotoxicity.

**Figure 1.**
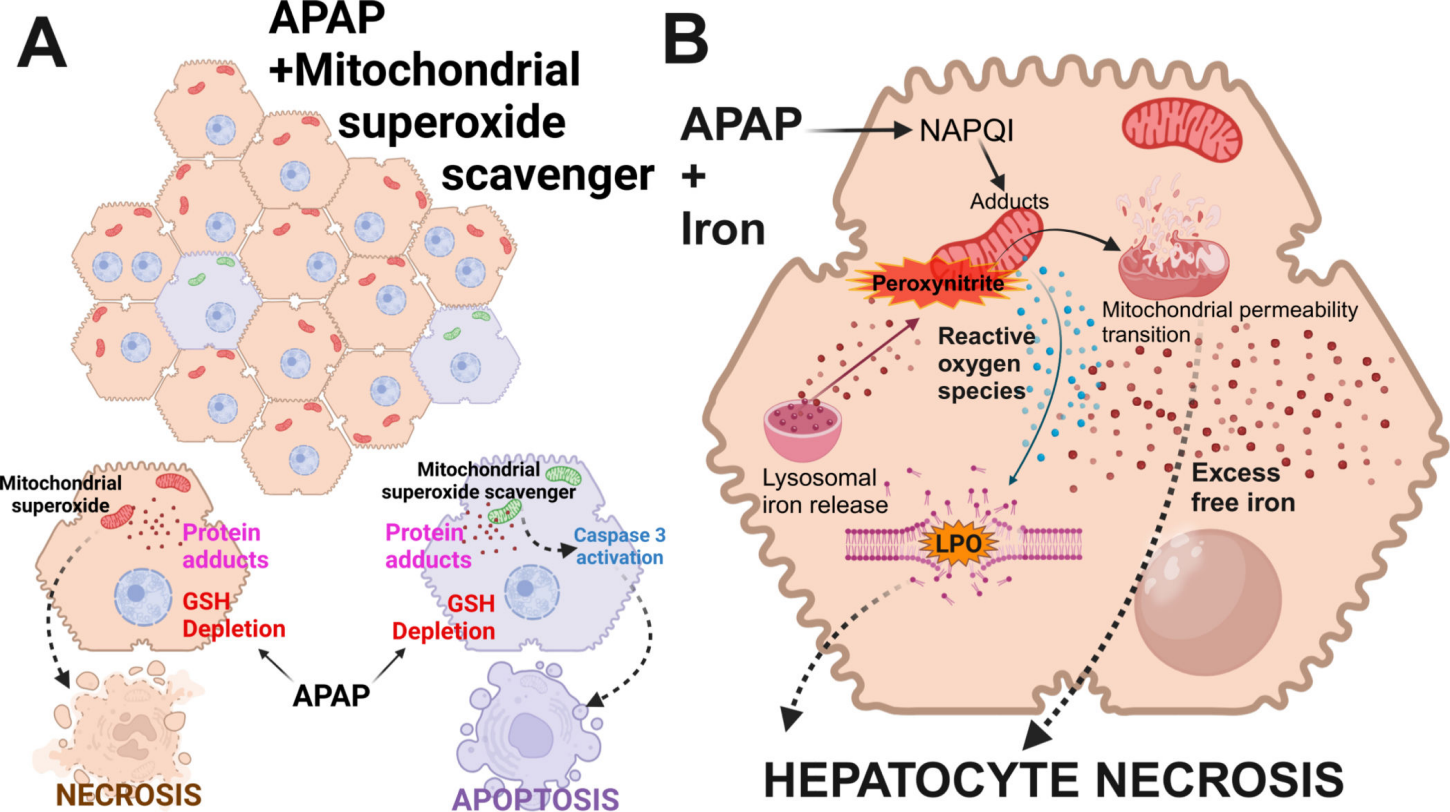
Alternate forms of cell death after APAP overdose. (**A**) APAP-induced cell death in hepatocytes typically occurs predominantly by necrosis. This is due to mitochondrial dysfunction after glutathione depletion, mitochondrial protein adduct formation, and mitochondrial oxidant stress. However, under conditions where mitochondrial superoxide production is inhibited, alternate pathways can activate caspase 3 cleavage to shift cell death to apoptosis in a subset of hepatocytes. (**B**) Under conditions of APAP overdose along with iron overload, the excessive cellular free iron reacts with mitochondrial reactive oxygen species (ROS) to induce lipid peroxidation (LPO) of membranes. This then contributes to hepatocyte necrosis along with the induction of the mitochondrial permeability transition. APAP: acetaminophen; GSH: glutathione. Adapted from [19]. © 2024 Authors. CC BY-NC 4.0. Created in BioRender. Ramachandran, A. (2025) https://BioRender.com/u09t107

In contrast to the absence of secondary necrosis, there is evidence for secondary apoptosis after APAP. This phenomenon was first described by Lemasters’ group [37] when APAP-induced MPTP opening and necrotic cell death in primary isolated hepatocytes was attenuated by MPTP inhibitors at early time points but not late time points where caspase activation was detected. In addition, the glycolytic substrate fructose and the membrane stabilizer glycine prevented the early necrosis but enhanced the late apoptosis [37]. This suggested that the primary mode of cell death in mouse hepatocytes is necrosis but if this signaling process is inhibited downstream at the mitochondrial level, secondary apoptosis can develop [37]. However, the initially assumed cause for the prevention of early apoptosis, namely declining ATP levels, could not be confirmed [55]. Although secondary apoptosis was thought to be an in vitro phenomenon for a long time, this effect was more recently observed in vivo. When animals were treated with the mitochondria-targeted MnSOD mimetic Mito-TEMPO, the prevention of peroxynitrite formation due to superoxide dismutation eliminated necrotic cell death 6 h after APAP administration with no evidence of apoptosis [29, 58]. Although the protection against necrosis was still present at 24 h, there was clear evidence of a limited number of apoptotic cells and caspase activation [58]. Consequently, co-treatment of Mito-TEMPO with a pancaspase inhibitor eliminated both necrosis and the secondary apoptosis [58]. Interestingly, promoting GSH synthesis with NAC treatment, which can affect early (NAPQI scavenging) and later events (scavenging of mitochondrial peroxynitrite) [32], attenuated necrotic cell death but did not induce secondary apoptosis [58]. Together, these observations suggest that if an intervention specifically targets later events in the necrotic signaling pathway, necrotic cell death can be prevented. However, the cellular stress of the upstream events, i.e., reactive metabolite formation, GSH depletion, and protein adducts formation, is still present and this stress may trigger apoptosis in a limited number of cells (Figure 1). This phenomenon may not be specific for APAP hepatotoxicity. Because these are time-dependent events, it is critical to always assess multiple time points in a pathophysiology and use parameters specific for apoptosis, while keeping in mind that many apoptosis parameters can also be modulated during a necrotic cell death.

## Is APAP-induced cell death caused by ferroptosis?

As mentioned earlier, though protein binding was recognized early as the initial step in APAP hepatotoxicity, mechanisms of subsequent free radical formation were controversial. While challenging the importance of the protein binding hypothesis, it was instead suggested that the key mechanism of cell death involves P450-derived ROS formation during APAP metabolism, which triggers extensive LPO [59, 60]. This caused a long-lasting controversy whether APAP-induced cell death is caused by protein adduct formation or by oxidant stress-derived LPO. However, it was not recognized at the time that evidence for the protein binding hypothesis was obtained in animals on a normal diet whereas the LPO hypothesis was derived from animals fed a vitamin E-deficient diet high in polyunsaturated fatty acids (PUFAs), i.e., in animals highly susceptibility to LPO [61]. Thus, data in support of each hypothesis was derived from different animal models, with mitochondrial oxidative stress being critical for APAP hepatotoxicity in mice on a normal diet, without appreciable LPO generation. However, the role of LPO in APAP toxicity has recently come into focus again with the identification of ferroptosis, which is a newly recognized iron-dependent form of cell death without mitochondrial dysfunction or bioenergetic failure [62]. The key components of this mechanism are oxidant stress, with ferrous iron-mediated Fenton reaction and LPO as the terminal events [62]. The process is facilitated by inhibition of cystine uptake by System Xc-, which reduces cellular GSH levels. This impairs the activity of glutathione peroxidase 4 (GPx4), which specifically reduces lipid hydroperoxides of PUFAs to the respective lipid alcohol and thereby interrupts the propagation of LPO [63]. In addition, any mobilization of iron through ferritinophagy (degradation of ferritin in lysosomes) or cellular iron uptake can enhance free iron levels in the cell. Furthermore, higher levels of PUFAs in cellular membranes due to diets high in unsaturated fatty acids enhance the susceptibility to LPO especially under conditions of vitamin E deficiency. Although the term “ferroptosis” is relatively new, the idea of iron-dependent LPO as a mechanism of cell death has been around for more than 60 years [19].

Extensive LPO as the cause of APAP and allyl alcohol-induced liver injury was first described by Wendel and coworkers [60, 61, 64–66]. They demonstrated an increase in specific LPO parameters such as ethane and pentane exhalation by 30- to-50-fold, accompanied by GSH depletion and loss of PUFAs (mainly 20:4 and 22:6); the liver injury was dependent on iron and could be inhibited by vitamin E [60, 61, 64–66]. Thus, this cell injury was consistent with what is now described as ferroptosis. However, the mice used for these experiments were on a diet deficient in vitamin E and high in soybean oil. In contrast, animals on a normal diet developed liver injury after APAP or allyl alcohol in the absence of relevant LPO [67–69], and vitamin E treatment did not protect [67]. Based on these findings, it was recognized for the first time that impairment of antioxidant defenses (vitamin E-deficiency) and enhanced levels of PUFAs (substrates for LPO) did not just amplify the injury mechanism but switched cell death signaling typically centered around mitochondrial dysfunction (in mice on normal diets) to one dominated by acute LPO [70]. However, vitamin E deficiency is a very rare condition in humans. Thus, liver injury caused by an APAP overdose does not involve LPO (ferroptosis) under normal conditions.

Progress in the understanding of the pathophysiology of APAP hepatotoxicity revealed that the key oxidant in mitochondria is peroxynitrite, a reaction product of superoxide and nitric oxide [71]. The critical role of peroxynitrite was documented by showing that mitochondria-targeted SOD mimetics eliminated nitrotyrosine protein adducts and injury [29, 72]; in contrast, a mouse partially deficient in MnSOD showed enhanced nitrotyrosine staining and dramatic aggravation of APAP-induced liver injury compared to a wildtype animal [73]. However, more recent studies assessing the role of ferrous iron in the pathophysiology first in vitro [74, 75] and then in vivo [76, 77] provided evidence that after a toxic dose of APAP ferrous iron derived from lysosomes is taken up into mitochondria through a Ca^2+^ uniporter and facilitates the MPTP opening and cell death. Ferrous iron can catalyze the formation of hydroxyl radicals through the Fenton reaction, which initiates LPO [78]. This would contradict the hypothesis of the role of peroxynitrite as the key oxidant in APAP pathophysiology and would also be inconsistent with the limited LPO. However, because the multistep chemical reaction of protein nitration by peroxynitrite requires the involvement of a metal ion such as ferrous iron [79], the role of iron in APAP hepatotoxicity is independent of the Fenton reaction under normal conditions [80]. Treatment with an iron chelator eliminated both protein nitration and liver injury in the absence of LPO [80], suggesting again that under normal conditions, APAP-induced cell death is unlikely to involve ferroptosis [80]. In support of these conclusions, the weak ferroptosis inhibitor ferrostatin-1 did not protect and the more potent inhibitor UAMC-3203 partially protected through off-target effects in the absence of relevant LPO [81].

When animals were co-treated with ferrous sulfate, APAP-induced liver injury was substantially enhanced, together with some increase in protein nitration [80]. However, the striking difference was a dramatic increase in LPO [80]; both the injury and LPO were almost completely prevented by an iron chelator [80] and partially reduced by the ferroptosis inhibitor UAMC-3203 [81]. In follow-up of these studies, it was shown that Mito-TEMPO, which is highly effective in protecting against APAP toxicity [29], did not reduce LPO or liver injury in animals co-treated with APAP and Fe^2+^ [82]. This suggests that the co-treatment with ferrous iron again switched the signaling mechanisms and mode of cell death from mitochondrial dysfunction-dependent necrosis after APAP alone to LPO-dominated ferroptosis after APAP + Fe^2+^ (Figure 1). Importantly, the dose of ferrous iron sulfate used in these studies was 0.15 mmol/kg FeSO_4_ (human equivalent dose: 1.85 mg/kg), which is less than the recommended dose for an iron supplement (1 tablet with 325 mg FeSO_4_ = 5.4 mg/kg). This raises the possibility that if a patient ingests some iron supplement tablets during a suicide attempt with an overdose of APAP [83, 84], the mechanism of cell death is switched to ferroptosis, and the accelerated and enhanced severe toxicity can be rapidly fatal [85]. Whether the standard antidote of NAC is less effective under these conditions remains to be evaluated. Taken together, under normal conditions, APAP-induced cell death involves peroxynitrite-mediated mitochondrial dysfunction but LPO and ferroptosis are unlikely to play a relevant role in the pathophysiology. However, if antioxidant defense systems are severely impaired or the more realistic scenario that an iron supplement is co-ingested, the mode of cell death can be switched to ferroptosis.

## Role of inflammation in APAP-induced liver injury and repair

The cellular necrosis after an APAP overdose leads to release of cell contents, which includes molecules termed damage-associated molecular patterns (DAMPs) that can bind to pattern recognition receptors, e.g., toll like receptors, on macrophages and trigger formation of various cytokines and chemokines [86–90]. These inflammatory mediators prime and activate inflammatory cells in circulation or in the bone marrow and recruit these cells into the liver. The main purpose of this sterile inflammatory response is to remove the necrotic cell debris and stimulate regeneration to restore liver function [87, 89, 90]. However, as seen in other forms of acute liver injury processes, e.g., hepatic ischemia-reperfusion injury, there is also the risk of aggravating the existing injury [91]. Hence, it is not surprising that there have been significant controversies around this topic over the years.

Kupffer cells, the resident macrophages of the liver, and monocyte-derived macrophages have both been implicated as cells that both contribute to the injury and are critical for regeneration. Early reports that Kupffer cell-induced oxidant stress and peroxynitrite formation may cause APAP-induced injury [92, 93] were questioned as the data could not be reproduced [94–96], NADPH oxidase-deficient mice were not protected [97, 98] and mitochondria in centrilobular hepatocytes were identified as the main source of superoxide and peroxynitrite under these conditions [72, 99]. On the other hand, Kupffer cell-derived IL-10 can restrict the injury by limiting iNOS induction [100, 101] and at later time points by promoting pro-regenerative gene expression in hepatocytes surrounding the area of necrosis [102]. A more detailed analysis of hepatic macrophages during APAP hepatotoxicity and recovery showed that there was a progressive loss of Kupffer cells during the injury phase with recovery during regeneration [103]. In addition, monocyte-derived macrophages are recruited into the liver during the injury phase mainly through Kupffer cell-generated monocyte chemoattractant protein 1 (MCP-1; CCL2) [104–107]. The newly recruited monocytes express a pro-inflammatory phenotype (Ly6C^hi^) [103] and there is evidence that during the very early phase of their recruitment (8–12 h after APAP) these cells can cause a temporary mild aggravation of the injury [108]. However, these cells convert rapidly to a pro-regenerative phenotype (Ly6C^lo^) [103] that is essential for recovery by removing cell debris and promoting regeneration [104, 105]. Thus, the critical role of monocyte-derived macrophages during recovery from APAP-induced liver injury is not controversial and there is also support for this mechanism in human livers [109]. However, the conversion of the recruited monocytes from the pro-inflammatory (M1) to the pro-regenerative (M2) phenotype is critical for regeneration. The signaling events involved are not completely understood but may involve ROS formation by neutrophils [110] or heme oxygenase-1-derived carbon monoxide [111]. These mechanisms are valid with moderate overdoses that lead to recovery; however, a more severe overdose can aggravate the injury and reduce and delay monocyte recruitment leading to impaired regeneration [107]. In addition to the removal of necrotic cell debris, monocyte-derived macrophages can also limit potential neutrophil toxicity by phagocytosis of these cells [105, 112]. At the end of the recovery phase, the remaining macrophages are removed by triggering apoptosis [113]. Thus, the experimental conditions determine the mechanism of monocyte recruitment, their function, and their clearance.

**Figure 2.**
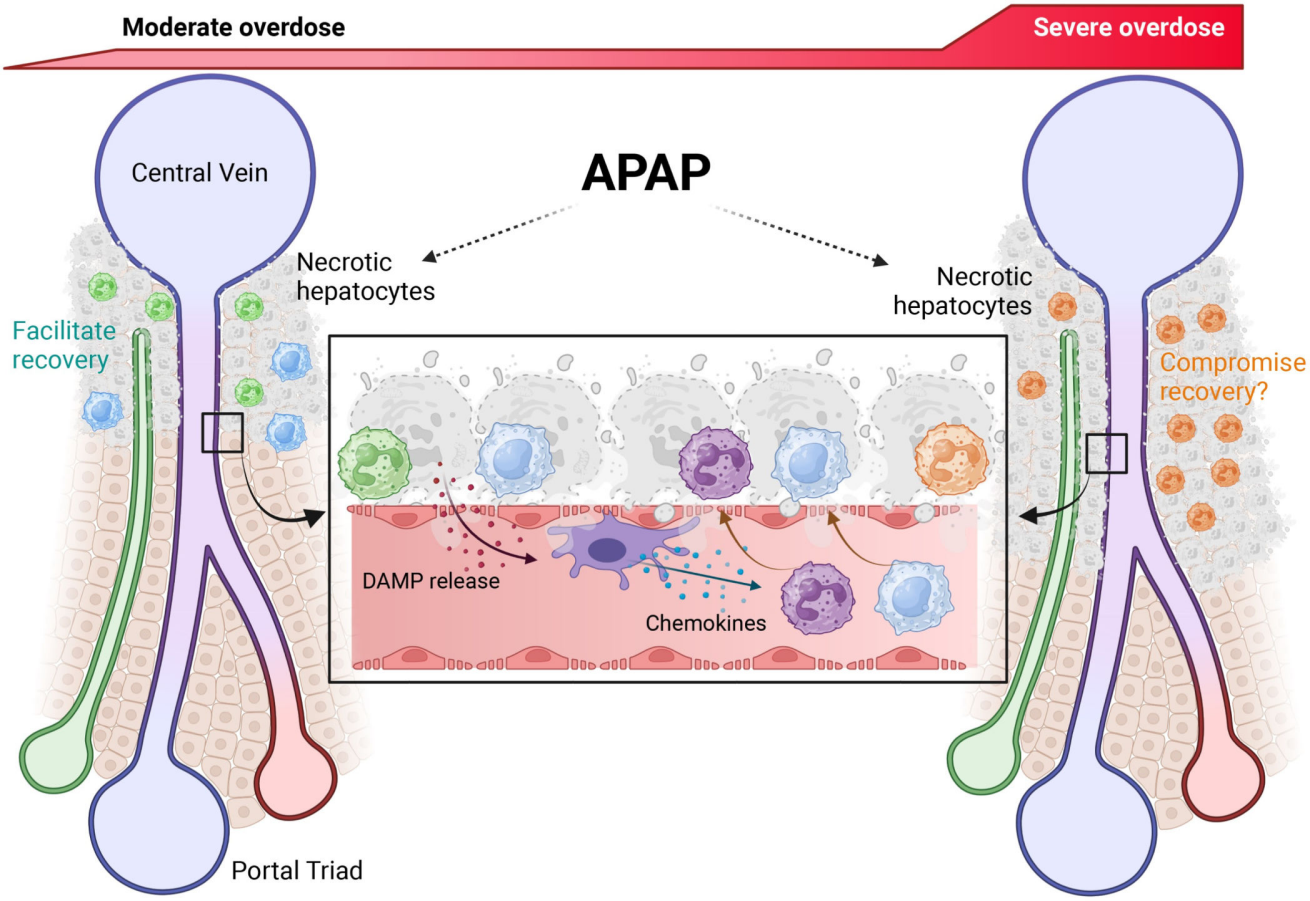
The sterile inflammatory response after moderate or severe APAP overdose-induced hepatocyte necrosis. APAP-induced necrosis of centrilobular hepatocytes necrosis releases damage-associated molecular patterns (DAMPs). These activate resident Kupffer cells within sinusoids, which release chemokines which attract neutrophils and monocytes towards the area of necrosis. These immune cells then transmigrate and infiltrate into the parenchyma where their subsequent behavior is dictated by the severity of the overdose. After a moderate overdose, infiltrating neutrophils and monocytes facilitate hepatocyte regeneration and liver recovery to regain homeostasis. However, the enhanced neutrophil infiltration accompanied by blunted macrophage response after a severe overdose likely compromises hepatocyte regeneration, thus preventing liver recovery. APAP: acetaminophen. Reprinted with permission from [122]. © 2025 Elsevier Inc.

Neutrophils are part of the innate immune system and are the first inflammatory cell type to be recruited into the liver during APAP hepatotoxicity [114]. Although neutrophils are known to aggravate liver injury in a variety of diseases [115], an early study using antibodies against adhesion molecules to block neutrophil cytotoxicity did not protect suggesting that neutrophils do not aggravate APAP-induced liver injury [114]. This conclusion was challenged when pretreatment with the neutropenia-inducing antibody Gr1 resulted in reduced liver injury [116]. However, posttreatment with anti-Gr1 did not protect [117], and pretreatment with anti-Gr1 caused a preconditioning effect with substantial activation of metallothionein 1 and 2 proteins [118]. Induction of these genes is known to be highly protective against APAP toxicity [119]. These and many other neutrophil-specific interventions (reviewed in [87, 90]) consistently did not protect against APAP hepatotoxicity, which confirmed the original observation that neutrophils do not aggravate APAP-induced liver injury [114]. More recently, additional studies provided direct evidence for a pro-regenerative role of neutrophils by enhanced phagocytosis of necrotic cell debris and by promoting the conversion of monocyte-derived macrophages to the M2 phenotype [110, 120]. Consistent with the data in mice, the activation of circulating neutrophils as an indicator for the activation status of hepatic neutrophils showed no neutrophil activation during the injury phase but substantial activation (ROS, phagocytosis) selectively during the recovery phase [98]. However, these patients all survived their serious liver injury and fully recovered. These human disease conditions are mimicked by treatment of fasted C57Bl/6J mice with the standard overdose of 300 mg/kg APAP, which causes liver injury followed by regeneration and recovery without mortality. However, when the experimental conditions are designed to reflect patients with severe liver injury, lack of regeneration, and acute liver failure, a dose of 600 mg/kg APAP must be used [121]. Under these conditions, the neutrophil response is dramatically aggravated and inhibition of hepatic neutrophil recruitment attenuated liver injury [107]. Thus, when a moderate overdose is used, the neutrophil response does not affect the injury but assists in the regeneration phase in both mice and humans. On the other hand, with higher overdoses, the neutrophil response can be amplified and can contribute to the injury (Figure 2). Under these conditions, monocyte recruitment and regeneration are delayed and attenuated leading to liver failure and mortality in mice and humans. It is therefore important to keep in mind the clinical scenario that is being mimicked, i.e., overdose causing injury and recovery versus a severe overdose with injury, limited recovery, and development of acute liver failure.

## Relevant factors that affect mechanisms and modes of cell death

### Species differences

It has been known for some time that there are species differences in the susceptibility of APAP toxicity [123]. For practical reasons, rats and mice are the main species used today for investigating mechanisms of APAP toxicity and evaluating therapeutic interventions. However, when comparing the injury after an overdose, only mice generally develop a similar degree of liver injury as observed in humans [123, 124]. Importantly, the mechanisms of toxicity in mice are very similar to humans [125, 126]. In contrast, most rat strains develop very limited injury even with much higher doses than used in mice [124]. However, the metabolic activation and protein adduct formation, also in mitochondria, are only marginally lower in rats compared to mice but there was no relevant mitochondrial dysfunction and oxidant stress in rats [124]. The reasons for this difference were detailed recently. Compared to humans and mice, rats have a higher basal level of antioxidant defense gene expression, and rats have a greater capacity to respond to cellular stress, which makes them generally more resistant to drug toxicity [127]. Thus, mice are more like humans in their stress response and are therefore better human-relevant models for APAP toxicity than rats.

### Mouse strains and substrains

There are many different mouse strains, which have a wide spectrum of susceptibility to APAP toxicity [128]. The C57Bl/6J strain, which is most often used for drug toxicity studies, has an average susceptibility to an APAP overdose [128]. Other popular strains such as Balb/c, C3Heb/FeJ, and B6C3F1/J mice develop more severe injury than C57Bl/6J mice after a 300 mg/kg dose of APAP [128]. However, these strains show more severe hemorrhage caused by sinusoidal endothelial cell (SEC) damage [99, 129], which may aggravate the hepatocellular injury due to microcirculatory disturbances leading to additional ischemic necrosis [95, 129, 130]. The variable susceptibility to hemorrhage is caused by the different expression levels of Cyp2E1 in SECs [129]. Balb/c mice express high levels of Cyp2E1 in SEC and show more severe liver injury and hemorrhage compared to C57Bl/6J mice with low expression of Cyp2E1 in SEC [129]. Because there is limited hemorrhage in human livers after an APAP overdose, the C57Bl/6J strain is more relevant for the human pathophysiology than mouse strains that develop extensive hemorrhagic shock [131].

Another issue is that the C57Bl/6 strain has 2 substrains, 6N and 6J, which have markedly different susceptibility to APAP [132]. The 6J substrain has a deficiency of the mitochondrial enzyme nicotinamide nucleotide transhydrogenase (NNT), which transfers electron from NADH to NADP^+^ thereby supporting the antioxidant glutathione peroxidase in mitochondria. However, under conditions of stress and inhibition of the electron transport chain, the enzyme works in the opposite direction by transferring electrons from NADPH to NAD^+^, which impairs mitochondrial antioxidant defenses and feeds more reducing equivalents into the impaired electron transport chain leading to increased electron leakage and superoxide formation [133]. Thus, the wildtype strain for NNT, the 6N substrain, generates more oxidant stress during APAP hepatotoxicity and suffers more severe liver injury compared to the NNT-deficient 6J substrain [132]. Although the general mechanisms of APAP-induced liver injury appear to be very similar in the 2 substrains [132], the risk for experimental design is that there is a mismatch in the substrains between WT and KO mice, which can lead to misinterpretation of the data. This happened when totally inconsistent results were published by several groups using JNK2-deficient mice [36, 134–136] and it was later discovered that the reason for the contradictory data was caused by the mislabeling of the background strain for JNK2 KO mice by the vendor [137], which led to the use of WT and KO mice with mismatched genetic background. To avoid these types of mishaps, the use of litter-mate controls is highly recommended.

### Hepatoma and other immortalized cell lines

Immortalized cell lines are popular tools to study drug hepatotoxicity because they are cost effective, readily available as cryopreserved cells, can be of human origin and generally show robust proliferation. However, major caveats include dramatic differences in the gene expression profile compared to primary hepatocytes [138]. This is a particular issue for drug metabolizing enzymes, especially cytochrome P450, which are substantially downregulated [139]. This reduces the susceptibility of the immortalized cells to drug toxicity and even switches the mode of cell death from necrosis in primary hepatocytes to caspase-dependent apoptosis in hepatoma cells [140, 141]. For example, HepG2 cells exposed to 20 mM APAP for 24 h showed only a minor (8%) reduction in GSH content, a minor increase in protein adducts, no mitochondrial dysfunction and only a 2-fold increase in lactate dehydrogenase (LDH) release [142]. In contrast, if metabolically competent human HepaRG cells [142] or primary human hepatocytes [46] were treated with 20 mM APAP for 24 h, there was 80–90% GSH depletion, a ten-fold higher protein adducts formation, 70–90% reduction of the mitochondrial membrane potential and 6–10-fold increase in LDH or ALT release. In addition, NAC treatment effectively protected primary human [46] and mouse hepatocytes [143] but not HepG2 cells against APAP toxicity [144]. Transfection of Cyp2E1 [145] or induction of Cyp2E1 by dimethyl sulfoxide (DMSO) [139], can restore at least in part the metabolic capacity of HepG2 cells but the overall gene expression profile of HepG2 cells shows low similarity to primary human hepatocytes [146, 147]. Thus, HepG2 cells and other immortalized cell lines should be used with caution. Even when tested for metabolic competency, the differences in the gene expression profiles compared to primary hepatocytes always raise concerns regarding the applicability of the investigated intracellular signaling mechanisms and the mode of cell death in humans.

### Adaptations and off-target effects

#### Gene knockout mice

Whole body or tissue-specific knockout of one or several genes is a frequently used approach in mechanistic research. Although it is generally considered a very specific approach to test the involvement of a certain gene in the pathophysiology, the approach is not without potential risks. Eliminating a gene from birth, may lead to adaptation that can affect the injury mechanism. For example, autophagy is a beneficial process that removes modified proteins and damaged mitochondria during APAP toxicity and limits the injury [148, 149]. On the other hand, inhibition of autophagy can aggravate APAP-induced liver injury [148–150]. Therefore, deficiency of a critical autophagy gene, e.g., *Atg5*, was expected to cause more severe injury after APAP overdose. However, in contrast to the expectation, *Atg5*-deficient mice did not develop liver injury after a toxic dose of APAP [151]. The reason for this unexpected observation was that paralysis of an essential cellular defense mechanism like autophagy leads to faster accumulation of cellular waste, which triggers an enhanced rate of cell turnover through apoptosis and compensated enhanced regeneration [151]. A secondary effect of this stress is activation of Nrf2 and its target genes resulting in compensatory higher GSH levels and induction of antioxidant genes in the livers of these mice, which makes these livers more resistant to additional acute stress such as APAP toxicity [151]. However, in the long-term, this gene deficiency leads to inflammatory injury, fibrosis, and ultimately liver cancer at later times [152]. One way to test for these types of effects is to compare a chronic gene deficiency with acute knockdown experiments and if the results are different, adaptive responses likely impacted the injury mechanism [153]. Thus, any experiment with gene knockout mice needs to consider adaptive mechanisms that can affect signaling mechanisms and the mode of cell death.

#### Chemical inhibitors and off-target effects

Another frequently used experimental approach is to use a chemical inhibitor to block an enzyme activity or otherwise induce or block the expression or the function of a gene. However, even the most specific inhibitor can have effects beyond the therapeutic target, i.e., off-target effects, which may significantly affect the outcome. Potential off-target effects can be suspected when the experimental results are opposite to what was expected but they are less likely pursued when the obtained results are consistent with the expectation. In this case, other cues may have to be paid attention to. For example, when investigating the hypothesis that gap junctions transport toxic metabolites to neighboring cells and support their killing during APAP and thioacetamide hepatotoxicity, the fact that the new gap junction inhibitor 2-aminoethoxydipenyl-borate (2APB) protected against the injury appeared to support the hypothesis [154]. However, the > 95% protection against both hepatotoxins with this inhibitor raised concerns that off-target effects might have played a role. Indeed, when the effect of 2APB on drug metabolism was evaluated, it was discovered that 2APB is a P450 inhibitor as directly shown in vitro and observed in vivo when the protection correlated with inhibition of protein adduct formation [155]. Interestingly, 2APB is also protected when administered after the metabolism phase by inhibition of JNK activation [155]. Together these mechanistic effects make 2APB are very potent antidote against APAP toxicity, which is likely totally independent of gap junctions as connexin 32 knockdown mice do not show consistent protection [156, 157]. Thus, in drug toxicity experiments, all chemicals administered as pretreatment or during the metabolism phase as posttreatment, need to be tested specifically for their effect on APAP metabolism.

**Figure 3.**
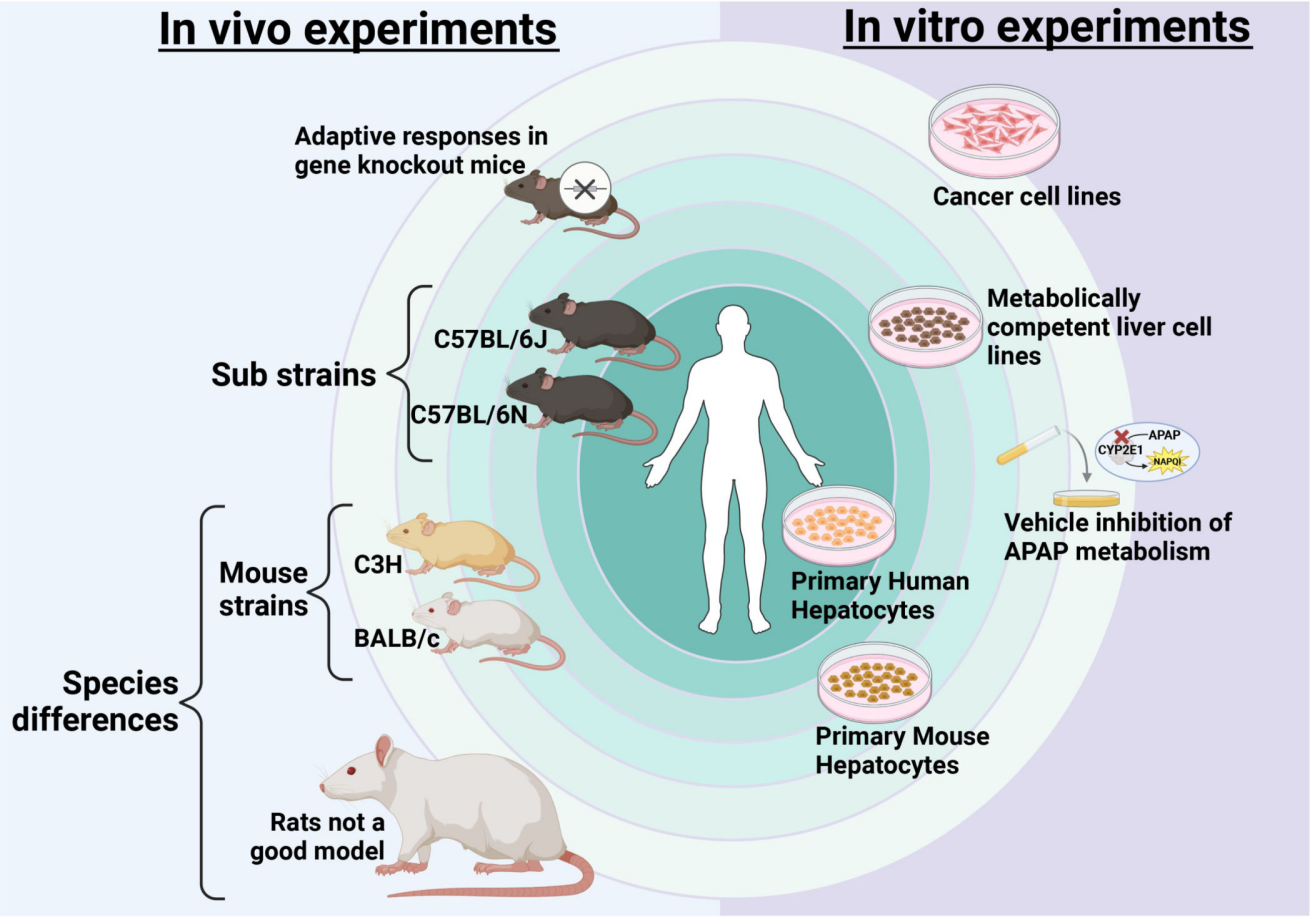
Factors influencing APAP pathophysiology. Several aspects of experimental design can influence mode of cell death and cell signaling in study of APAP hepatotoxicity. The ultimate aim of any experimental system should be replication of human pathophysiology which is central to translate findings to the clinic. For in vivo experiments, the closest model would be the C57BL/6 mouse strain though degree of liver injury can vary between sub-strains without change in mechanism. Other mouse strains such as C3H or BALB/c would be the next option though some aspects of human pathophysiology may not be replicated in these strains. The furthest from replicating human pathophysiology would be other species such as rats, which are not a good model for APAP hepatotoxicity. Additionally, adaptive responses in gene knockout mice could also skew responses which would need to be considered. For in vitro experiments, primary human hepatocytes would be the optimum model closely followed by isolated primary mouse hepatocytes. Next would be metabolically competent human liver cell lines. However, cancer cell lines would not be a good model due to lack of metabolic competency and effects of vehicles or interventions on APAP metabolism also need to be considered. APAP: acetaminophen. Created in BioRender. Ramachandran, A. (2025) https://BioRender.com/w79i025

Another frequently encountered problem using chemical inhibitors is the choice of solvent. One of the most popular solvents for compounds with limited water-solubility is DMSO. However, DMSO is also a potent inhibitor of cytochrome P450 [158, 159]. Thus, any compound that requires DMSO as solvent or at least contains a significant amount of DMSO to solubilize it needs to be injected outside the metabolism phase of APAP, which is about 1.5–2 h for a 300 mg/kg dose and longer with higher doses. It is also critical to include a solvent control in the experimental design, which does not mean a group treated with DMSO alone but an APAP + DMSO group. In the case of the JNK inhibitor SP600125, which is only soluble in DMSO and requires early treatment, 70% of the protection against APAP toxicity was caused by DMSO and 30% by SP600125 [36]. Of note, under these conditions, higher doses of APAP (500 mg/kg) are required to achieve liver injury [36]. Only more recently it was shown that a water-soluble JNK inhibitor can be responsible for 100% of the protection [160]. Furthermore, caspase inhibitors generally require DMSO as a primary solvent. Ignoring the solvent control, it was claimed that pretreatment with the pancaspase inhibitor Z-VAD-fluoromethylketone protected against APAP toxicity despite lack of caspase activation [41]. This contradiction was reconciled by showing that the protection was caused exclusively by the solvent DMSO and not the caspase inhibitor [161]. Furthermore, the water-soluble caspase inhibitor Ac-DEVD-CHO did not protect against the liver injury [162]. Another example was a study showing the involvement of natural killer (NK) cells and NK T-cells in the pathophysiology of APAP toxicity [163]. However, as the results could not be reproduced by other groups [164], it was finally realized that the authors of the original study dissolved APAP in DMSO instead of saline. When the experiments were repeated with APAP dissolved in saline versus DMSO, it was shown that the presence of DMSO activated NK T-cells and led to a role of these immune cells in the pathophysiology [164]. However, in the absence of DMSO, NK, and NK T-cells are not involved in APAP hepatotoxicity. Together, these examples show that with every use of chemical interventions off-targets for both the chemical and the solvent need to be ruled out for the proper interpretation of the results and mechanistic conclusions (Figure 3).

## Translation of experimental studies to patients

Although there is extensive evidence that mechanisms of APAP hepatotoxicity translate to human overdose patients, the vast majority of experiments are performed in young and healthy male mice, which does not reflect the entire spectrum of patients in terms of age, sex, pre-existing conditions, consumption of multiple drugs, etc. In terms of sex, female mice were shown to be less susceptible to APAP toxicity [165–167]. The mechanisms of cell death are the same in male and female mice but the enhanced recovery of hepatic GSH levels in female mice attenuates the injury [167]. However, after higher overdoses of APAP, no difference in injury was observed between male and female mice [107]. Importantly, all studies in humans [45, 168–170] and in primary human hepatocytes [46] involved both male and female patients or donors, respectively, with no obvious differences in mechanism.

Another factor overlooked by using young mice is that age may affect toxicity. However, the age distribution of patients with self-harm intent indicates a substantial peak between 15 and 24 years of age with the lowest number of cases in the > 50-year age group [4, 171]. In contrast, unintentional overdosing appears to be relatively equally distributed between all age groups [4, 171]. Thus, using younger mice replicates the clinical scenario for a large group of patients. Although old age is associated with increased utilization of APAP, mouse studies did not find increased hepatotoxicity after acute, chronic, and sub-acute APAP exposure in old mice [172]. It appears that old age per se is not a risk factor for APAP toxicity, however, co-morbidities with impaired liver function, low body weight, polypharmacy, etc. can be risk factors that increase with age, leading to higher susceptibility to adverse drug reactions [173]. However, comorbidities like compensated cirrhosis did not cause any adverse effects at least in response to therapeutic doses of APAP [174].

Another risk factor for APAP toxicity is ethanol. The basis for the enhanced toxicity is that chronic ethanol exposure can induce Cyp2E1 enzyme activities and thus enhance NAPQI formation [175]. In contrast, during acute ethanol exposure, ethanol competes with APAP for Cyp2E1 metabolism, which reduces NAPQI formation as long as ethanol is present and attenuates liver injury [176]. Thus, when using alcohol models, strict controls are necessary to avoid misinterpretation of the data. In human studies, chronic alcohol exposure did not cause toxicity after maximal therapeutic doses for several days [177]. One aspect that needs to be considered is that in these volunteer studies, patients are being fed an adequate diet during the trial. Thus, these studies suggest that ethanol alone is not a serious risk factor for APAP toxicity at therapeutic doses for most patients [178]. On the other hand, chronic alcohol abuse in combination with malnutrition (low hepatic GSH levels), longer duration of supratherapeutic APAP consumption, and older age with potential co-morbidities can trigger hepatotoxicity [179]. The challenge is to develop animal models that accurately account for these variables relevant to alcoholic patients. The results and mechanisms can vary considerably dependent on the experimental design.

Another potential risk factor that may be present in an increasing number of patients is hepatic steatosis or steatohepatitis caused by obesity, type 2 diabetes, and poor diet [180]. While various risk factors for enhanced APAP toxicity have emerged in the numerous experimental models of steatosis, including induction of Cyp2E1, reduced phase II metabolism, mitochondrial dysfunction, etc., opposite effects have also been described, suggesting that steatosis may not affect toxicity [180]. These inconsistent results also translate to human studies where obesity and steatosis correlated with increased hepatotoxicity after an APAP overdose in some studies [181] and no correlation of toxicity with obesity was observed in other studies [182]. These variable results both in experimental studies and in humans make it difficult to decide which models and experimental conditions are most relevant for the human pathophysiology in the context of steatosis and APAP hepatotoxicity.

A recent controversial topic is whether exposure to therapeutic doses of APAP during pregnancy can cause problems with neurodevelopment. Most of the evidence for this hypothesis comes from retrospective epidemiological studies [183] although these data are not conclusive [184]. Importantly, there is very little direct experimental evidence in support of this hypothesis, which is based on the assumption that the neurotoxicity is caused by similar mechanisms as described for hepatotoxicity [183]. However, this extrapolation from one organ to another is questionable as the exposure levels, the phase I metabolic capacity to generate NAPQI, the phase II metabolic capacity to eliminate APAP, and potential adaptive mechanisms in response to an APAP-induced cellular stress are not well described in the CNS system. A recent study showing that an APAP overdose triggers kidney injury through apoptosis and not necrosis as in the liver [162], should be a warning against extrapolating mechanisms of cell death studied in the liver to other organs. A case in point, when 2 different mouse strains were exposed to therapeutic or hepatotoxic doses of APAP, depletion of GSH or APAP protein adduct formation was only detected in the liver but not in the whole brain or individual brain regions, suggesting that there is no evidence for the formation of the reactive metabolite NAPQI in the brain of adult mice [185]. These observations are consistent with the absence of APAP protein adducts in the brain after even higher overdoses [186]. These findings question whether even after severe toxic doses of APAP, reactive metabolites can be formed in the brain, let alone after a therapeutic dose as taken during pregnancy. And this does not even consider that drug metabolizing enzymes in the fetus or newborn are generally lower than in adults.

## Conclusions

The assumption that there can only be one signaling mechanism of cell injury and one mode of cell death for a given insult such as APAP overdose is clearly incorrect. Although many contradictory results and conclusions can be attributed to lack of understanding of the established mechanisms, methodological problems and misinterpretation of data, there are many legitimate ways the signaling mechanism of injury can be affected resulting from minor modifications up to major changes including a complete switch of the mode of cell death. When investigating injury mechanisms and testing potential therapeutic interventions, it is therefore important to be mindful of the experimental design that could influence results including use of the most appropriate species and strains, optimal doses of APAP, the best experimental conditions (fasted vs fed, etc.), awareness that any treatment or interventions can have off-target effects including effects on the drug metabolism and antioxidant defense systems and be cognizant of the fact that genetic manipulations can trigger adaptive responses that also influence the outcome. Most importantly, it is essential for mechanistic insight that is supposed to have translational potential to keep in mind the clinical relevance of the experimental design and how the data generated can be reconciled with other published reports. Appreciation of these issues will determine whether the results of an experiment are relevant for understanding mechanisms of cell death and identification of novel therapeutic targets for most patients or if they are clinically irrelevant and just adding background noise to the literature.

## Data Availability

Not applicable.

## Abbreviations

2APB: 2-aminoethoxydipenyl-borate
APAP: acetaminophen
DAMPs: damage-associated molecular patterns
DMSO: dimethyl sulfoxide
GSH: glutathione
LDH: lactate dehydrogenase
LPO: lipid peroxidation
MPTP: mitochondrial membrane permeability transition pore
NAC: *N*-acetylcysteine
NK: natural killer
NNT: nicotinamide nucleotide transhydrogenase
P-JNK: phosphorylation of c-jun N-terminal kinase
PUFAs: polyunsaturated fatty acids
ROS: reactive oxygen species
SEC: sinusoidal endothelial cell
